# Sanitary Control

**Published:** 1899-12

**Authors:** 


### SANITARY CONTROL.

THE important move of Rochester, N. Y., will be a step followed soon by every other large city in the country, and soon statistics will prove its value of so much moment that a general appreciation in the price of milk will follow, of no burden to the people, and which will afford the producer revenue sufficient to surround the production of his milk with all of these great safeguards.

An ordinance relating to the keeping and sale of milk in the city of Rochester :

Section 1. No milk shall be sent into the city of Rochester, nor shall milk produced in the city of Rochester from cows maintained in the city of Rochester, be offered for sale in said city for any purpose whatsoever unless there shall be presented to the Secretary of the Board of Health a certificate or bill of health for each and every cow-stable where such milk shall have been produced, from a veterinarian, a graduate of an organized school of veterinary medicine, and duly licensed under the laws of New York, which said certificate shall show the following facts :

First. The name of the owner of the cow or herd.

Second. That the cows are in good physical condition and free from infectious diseases.

Third. That within three months last past they have been tested by tuberculin, which tuberculin test has been applied under the latest and most approved methods, and that such tuberculin test has shown said cows and each thereof to be free from tuberculosis.

Fourth. Whether said cows or any thereof are afflicted with other infectious or contagious disease.

Fifth. Their general physical condition.

Sixth. A description of the cow tested, by breed, grade, color, markings, or other descriptive features.

Seventh. A table showing the temperature of the cows, just before and just after the test, including the individual temperatures and the temperature taken every two or three hours, beginning seven or eight hours after the injection, and taken every two or three hours until five or six temperature readings have been taken two or three hours apart.

No cows shall be added to any herd of cattle so certified to until it has been subjected to the tuberculin test, and a certificate as above required, duly filed in the office of the health department. Every cow and every herd of cattle from which milk is produced for use in the city of Rochester shall be subjected to the tuberculin test not more than one year from the date of the primary examination by tuberculin as required by this ordinance, and a new certificate or bill of health as required by this ordinance shall be furnished to the Secretary of this Board. Every certificate should be sworn to before a notary.

Where a cow furnishing milk for human consumption in the city of Rochester, or where one or more cattle of a herd furnishing milk for human consumption in the city of Rochester, shall be found upon examination by the tuberculin test to be affected with tuberculosis, it shall be the duty of the veterinarian making the examination to immediately report the case or cases to the secretary or executive officer of this Board.

Section 2. Any person, firm, or corporation violating any of the provisions of this ordinance shall be subject to a penalty of fifty dollars.

Section 3. No person, firm, or corporation shall have in his or its possession for sale, or shall offer for sale in the city of Rochester, any unclean milk; and for the purpose of determining the standard of cleanliness of milk, it is hereby declared that in any sample of milk found to contain more than 100,000 bacteria per cubic centimetre the said milk is declared to be unclean, unfit for use, and dangerous to health. Any person, firm, or corporation violating the provision of this section shall be subject to a penalty of fifty dollars.

Section 4. This ordinance shall take effect on and after the first day of October, 1899.

